# Periodontitis and atherosclerotic cardiovascular disease

**DOI:** 10.1016/j.mocell.2024.100146

**Published:** 2024-11-06

**Authors:** June Yeon Kim, Kyeongho Lee, Moon Geon Lee, Sung-Jin Kim

**Affiliations:** Department of Oral Histology and Developmental Biology, School of Dentistry and Dental Research Institute, Seoul National University, Seoul, Korea

**Keywords:** Atherosclerotic cardiovascular disease, Causal relationship, Mendelian randomization, Periodontitis, Systematic review

## Abstract

Atherosclerotic cardiovascular disease (ASCVD) is a major global health concern linked to significant morbidity and mortality. Recent research has explored the relationship between ASCVD and periodontitis, a prevalent inflammatory oral condition. Epidemiological studies have suggested a strong association between periodontitis and ASCVD, even proposing that periodontal disease could be a modifiable risk factor for cardiovascular conditions. This review critically analyzes the current evidence for a potential causal role for periodontitis in ASCVD. While randomized controlled trials have demonstrated reductions in surrogate markers of cardiovascular risk following periodontal interventions, these studies remain inconclusive regarding their direct effects on cardiovascular events. Preclinical studies in animal models have suggested a potential causal relationship between periodontitis and ASCVD, proposing several biological mechanisms to explain this connection. These studies, however, are limited in their ability to definitively prove causality. The positive associations observed in epidemiological studies between periodontitis and ASCVD may also be influenced by various biases, such as confounding and collider stratification. Moreover, our systematic review of Mendelian randomization studies on the causal relationship between periodontitis and ASCVD found no evidence of a genetic causality, further challenging the causal hypothesis. This review underscores the need for further high-quality research clarifying the relationship between periodontitis and ASCVD to better guide clinical practice and public health policy.

## INTRODUCTION

Atherosclerotic cardiovascular disease (ASCVD) encompasses various cardiovascular conditions associated with the buildup of atherosclerotic plaque in arterial walls, including coronary artery disease (CAD), stroke, and peripheral artery disease (Virani et al., 2020). ASCVD is a leading cause of global mortality, contributing to approximately one-fifth of total deaths (Martin et al., 2024). To address this, extensive research has delved into ASCVD risk factors in the hope of enhancing prevention, prediction, and management strategies. While the nonmodifiable risk factors for ASCVD include age, gender, family history of premature ASCVD, genetics, and ethnicity/race, the modifiable factors include hypertension, diabetes, hypercholesterolemia, and smoking. However, because these known risk factors do not seem to fully account for all ASCVD cases, considerable work remains in the search for unknown risk factors (Wong et al., 2022).

Periodontitis is a common inflammatory condition that compromises dental support tissues and affects 20% to 50% of the global population (Chen et al., 2021). Periodontitis is increasingly acknowledged not only as a threat to oral health but also as a risk factor for various noncommunicable diseases. Recent studies have reported an association between periodontitis and ASCVD (Carra et al., 2023), suggesting that improved management of periodontitis may reduce the risk of ASCVD. As periodontitis can often be managed effectively, this could prove highly significant in reducing the global ASCVD burden. Still, establishing a causal relationship between periodontitis and ASCVD remains challenging due to the ethical and practical constraints of conducting long-term randomized controlled trials (RCTs). Despite conflicting evidence, numerous studies have investigated this relationship. In this review, we critically evaluate recent evidence concerning a potential causal relationship between periodontitis and ASCVD.

## THE EPIDEMIOLOGICAL ASSOCIATION BETWEEN PERIODONTITIS AND ASCVD

Many epidemiological studies have investigated the association between periodontitis and ASCVD. In a series of systematic reviews (Carra et al., 2023, Dietrich et al., 2013, Herrera et al., 2020), researchers examined 32 epidemiological studies conducted from 1998 to 2022, finding that 28 of them (16 on prevalent ASCVD and 12 on incident ASCVD) suggested a positive association between periodontitis and ASCVD. Together, these studies support a significant association between periodontitis and ASCVD. Among the 4 studies that yielded negative results, Dain et al. (2021) conducted a cross-sectional study, while Tuominen et al. (2003), Gao et al. (2021), and Winning et al. (2020) conducted prospective cohort studies. Notably, Winning et al. (2020) reported an association between periodontitis and prevalent CAD but found no association with incident CAD. While these reports suggest a positive association between periodontitis and ASCVD, further research is necessary to validate the association with incident ASCVD.

## EVIDENCE FOR A CAUSAL RELATIONSHIP BETWEEN PERIODONTITIS AND ASCVD

### Effects of Periodontal Treatment on ASCVD or Its Parameters

The best way to investigate a potential causal relationship between periodontitis and ASCVD would be to perform an RCT that induces periodontitis in randomly allocated participants. This is impossible for practical and ethical reasons. Instead, it is possible to conduct an RCT investigating the effect of periodontal intervention in patients with existing periodontitis. Still, even in such a study, refusing to treat a patient would be ethically unacceptable. Therefore, the “no treatment group” often comprises patients forced to delay periodontal interventions during the clinical trial or community treatment protocol in which patients are managed by their general dental practitioner. This inevitably limits the study’s follow-up period.

Due to limited follow-up periods, most clinical studies have observed the influence of periodontal treatment on surrogate markers of cardiovascular disease rather than on disease incidence itself. For example, periodontal interventions reduced inflammatory mediators and thrombotic/hemostatic markers such as C-reactive protein (CRP), fibrinogen, white blood cell counts, cytokines, flow-mediated dilatation, and systolic blood pressure (Carra et al., 2023, D'Aiuto et al., 2013, Herrera et al., 2020). The improvement of these surrogate markers, however, does not necessarily translate to a reduction in cardiovascular events, as these events often arise from long-term chronic conditions.

Ye et al. (2022) conducted a systematic review of RCTs examining the preventive impact of periodontal treatment on ASCVD events. They included 2 studies meeting their criteria with over 1 year of follow-up. The first study by Lopez et al. (2012) focused on primary prevention in patients with metabolic syndrome and periodontitis. Participants in the experimental treatment group (*n* = 82) received supra-/subgingival scaling and root planing plus metronidazole and amoxicillin, while participants in the control treatment group (*n* = 83) received supragingival scaling plus 2 placebo tablets. By the time of a 12-month follow-up visit, the experimental group had suffered 4 cardiovascular events, while the control group had suffered none. The second study referred to as the PAVE pilot study by Beck et al. (2008) explored secondary prevention in patients with a history of ASCVD events. The intervention group (*n* = 151) received periodontal treatment, while the community care group (*n* = 152) received oral hygiene instructions. The incidence of cardiovascular events did not significantly differ between groups, but Ye et al. (2022) deemed the results inconclusive due to variable follow-up periods and high loss to follow-up. Therefore, they concluded that there is insufficient evidence to support the effect of periodontal treatment on primary or secondary prevention of CVD in individuals with periodontitis and ASCVD. In summary, there is sufficient evidence to conclude that periodontal intervention reduces the surrogate markers of ASCVD but insufficient evidence to conclude that it prevents ASCVD events.

### Preclinical Models

Preclinical studies using animal models can also provide insight into the relationship between periodontitis and ASCVD. Periodontitis can be induced in mice using various methods, such as placing ligatures around the teeth or injecting specific periodontal bacteria species or their lipopolysaccharides (Rojas et al., 2021). In contrast to the infeasibility of such studies in humans, these animal models also make it possible to investigate the effect of periodontitis on the incidence of cardiovascular disease.

The induction of periodontitis in mouse models promotes the development of atherosclerosis. Notably, ligature-induced periodontitis, one of the most common methods for inducing periodontitis, accelerated the formation of atherosclerotic plaque in hyperlipidemic apolipoprotein E-deficient (*Apoe*^null^) mice (Suh et al., 2022). Similarly, the induction of periodontitis by inoculating periodontal pathogens such as *Porphyromonas gingivalis* (*P. gingivalis*), *Treponema denticola* (*T. denticola*), and *Fusobacterium nucleatum* (*F. nucleatum*) also increased atherosclerotic plaque formation (Chukkapalli et al., 2014, Velsko et al., 2014, Zhou et al., 2022). These findings strongly suggest a causal relationship between periodontitis and ASCVD in mouse models.

Still, establishing a causal relationship between periodontitis and ASCVD in animal studies requires careful interpretation. These models often induce extreme inflammatory conditions that do not fully represent human pathophysiology. For instance, ligature-induced periodontitis in mouse models can cause severe alveolar bone destruction within just 1 week (Kittaka et al., 2023), a rate of progression far exceeding what is typically observed in humans. Such accelerated and intense inflammatory responses in animal models may not accurately reflect the chronic and gradual nature of human periodontitis. Moreover, oral inoculation of pathogenic bacteria likely leads to a more severe bacteremia than that observed in human periodontitis patients. This may exaggerate the impact of periodontitis on ASCVD onset in animal models, especially because bacteremia itself—induced by intravenous or intraperitoneal injection of *P gingivalis*—accelerated atherogenic plaque progression in atherosclerotic mouse models (Ford et al., 2007, Li et al., 2002).

### Biological Possibility

**Fig. 1:**
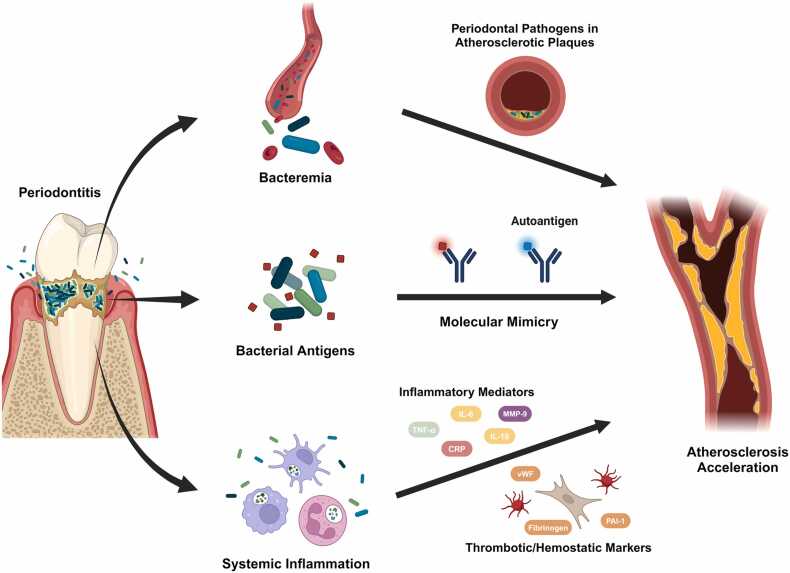
Biological mechanisms explaining a potential causal role for periodontitis in ASCVD. Periodontitis leads to bacteremia as oral bacteria enter the bloodstream through ulcerated periodontal pockets, especially following oral hygiene practices or clinical procedures. These bacteria can reach atherosclerotic plaques, where they exert harmful effects. Bacterial antigens that resemble host autoantigens may trigger an autoimmune response through molecular mimicry, contributing to plaque formation and progression by activating endothelial cells, monocytes, and T cells. Additionally, periodontal infections elevate systemic inflammatory mediators, including CRP, TNF-α, interleukins, and MMP-9, as well as thrombotic markers such as plasminogen activator inhibitor-1, fibrinogen, and vWF, thereby accelerating atherogenesis.

There are several possible biological mechanisms through which periodontitis could contribute to ASCVD. Patients with periodontitis often have ulcerated periodontal pockets accompanied by gingival bleeding, providing oral bacteria access to the bloodstream and leading to bacteremia. Since mechanical irritations can promote bacterial entry into the bloodstream via gingival bleeding, many studies have examined their potential to induce bacteremia. A systematic review and meta-analysis of 12 studies indicated an increased prevalence of bacteremia following toothbrushing (Tomas et al., 2012). Another systematic review showed that nearly half (49.4%) of patients exhibited bacteremia after periodontal procedures (Horliana et al., 2014). These findings suggest bacteremia is readily triggered in periodontitis patients through routine oral care practices or therapeutic interventions (Fig. 1).

Other evidence supports the potential contribution of periodontal bacteria to the development of atherosclerotic plaques. A meta-analysis of 63 studies confirmed the presence of 23 oral commensal bacteria in the atherosclerotic plaques of CAD patients (Chhibber-Goel et al., 2016). *In vitro* experiments demonstrated that periodontal bacteria, including *P gingivalis, Aggregatibacter actinomycetemcomitans (A. actinomycetemcomitans), and F nucleatum* can invade human cardiovascular cells, such as endothelial and smooth muscle cells (Reyes et al., 2013). Furthermore, unlike the wild-type *P gingivalis* strain, mutant strains with significantly reduced invasive ability *in vitro* failed to accelerate atherosclerosis in *Apoe*^null^ mice (Gibson et al., 2004). These findings suggest periodontal bacteria may reach atherosclerotic plaques and invade cardiovascular cells, contributing to atherosclerotic plaque development.

In another possible mechanism, the penetration of bacteria or their lipopolysaccharides into periodontal lesions may elevate systemic inflammation and promote or exacerbate atherogenesis. Numerous studies have reported increased levels of inflammatory mediators, such as CRP, interleukins, amyloid A, MMP-9, and platelet-activating factor, in the blood of patients with periodontal disease (Herrera et al., 2020, Schenkein and Loos, 2013). Since atherosclerosis is a chronic inflammatory condition primarily driven by the innate immune response of myeloid cells like monocytes and macrophages (Wolf and Ley, 2019), the rise in systemic inflammatory mediators due to periodontal infections may accelerate atherosclerotic plaque formation. Studies have also noted elevated levels of thrombotic and hemostatic markers, such as fibrinogen, plasminogen activator inhibitor-1, von Willebrand factor, and selectins, in periodontitis patients (Herrera et al., 2020, Schenkein and Loos, 2013). These factors are closely associated with vascular inflammation and play a crucial role in atherogenesis and thrombosis by promoting platelet aggregation and thrombus formation (Davalos and Akassoglou, 2012, Popovic et al., 2012).

Periodontal microorganisms can also influence the progression of atherosclerosis through a mechanism known as “molecular mimicry.” This occurs when the structure of a foreign antigen closely resembles that of the host's self-antigen, leading to the production of antibodies inducing an autoimmune response (Kohm et al., 2003). For example, heat shock protein 60 (HSP60) is highly conserved in both prokaryotic and eukaryotic cells. Periodontal pathogens such as *A actinomycetemcomitans, P gingivalis, T denticola,* and *F nucleatum* express GroEL, a bacterial ortholog of human HSP60 (Maeda et al., 1994, Vayssier et al., 1994). Elevated levels of antibodies against *P gingivalis* GroEL and *F nucleatum* GroEL have been observed in periodontitis patients (Lee et al., 2012, Ueki et al., 2002). In addition, GroEL-specific T-cell lines derived from atherosclerotic plaques showed cross-reactivity to human HSP60, suggesting molecular mimicry between GroEL and human HSP60 (Ford et al., 2005).

The cardiolipin/β2-glycoprotein I (β2GPI) complex may also play a role in molecular mimicry. β2GPI is a plasma protein with a high affinity for binding negatively charged phospholipids like cardiolipin, which is a unique phospholipid found primarily in the inner mitochondrial membrane. β2GPI circulates freely in the blood, binding to exposed cardiolipin on damaged cells. Several periodontal pathogens present antigens that resemble cardiolipin or β2GPI, and periodontitis patients can show elevated levels of anticardiolipin and anti-β2GPI antibodies (Schenkein et al., 2003, Wang et al., 2008). This molecular mimicry triggers an autoimmune response, increasing the risk of atherosclerosis by damaging endothelial cells, promoting foam cell formation, or inhibiting the protective, antiatherogenic effects of certain molecules (Schenkein and Loos, 2013).

Despite the insights these findings have provided, limitations remain in their support for a causal relationship between periodontitis and ASCVD. For example, bacteremia caused by periodontal disease is typically short-lived, being quickly cleared by the immune system. It remains unclear whether transient bacteremia alone can lead to the chronic inflammation and vascular damage observed in ASCVD. Moreover, there is insufficient evidence that periodontal pathogens consistently colonize or infect arterial walls. Regarding systemic inflammation, the increase in inflammatory markers associated with periodontal disease is modest compared with other chronic inflammatory conditions, such as rheumatoid arthritis or chronic kidney disease, and the causal nature of the relationship between these conditions and ASCVD is also controversial. Finally, it remains unclear whether molecular mimicry acts as a harmful stimulus for the immune system or instead contributes to immune tolerance (Rojas et al., 2018). Given these uncertainties, further research is needed to provide direct evidence of the contribution of these biological mechanisms to ASCVD.

## EVIDENCE AGAINST A CAUSAL RELATIONSHIP BETWEEN PERIODONTITIS AND ASCVD

### Mendelian Randomization

**Fig. 2:**
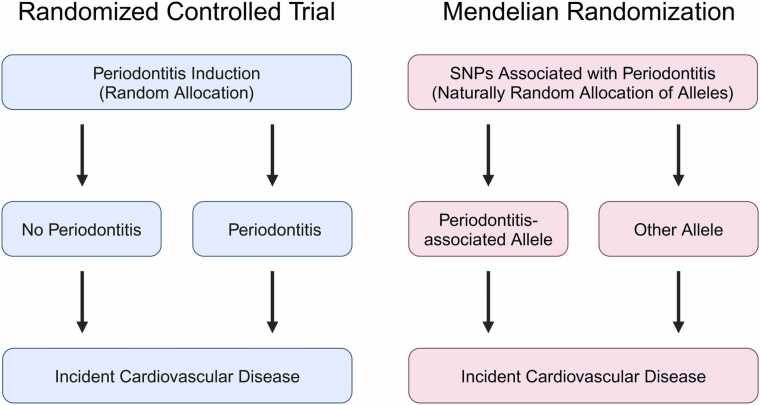
Schematics comparing randomized controlled trials and Mendelian randomization studies. The left panel illustrates a randomized controlled trial (RCT) where individuals are randomly allocated to either a periodontitis induction group or a no-periodontitis group, followed by the observation of incident cardiovascular disease. The right panel depicts a Mendelian randomization approach, where individuals are naturally allocated according to the presence of alleles associated with periodontitis or other alleles, and the incidence of cardiovascular disease is observed. This method leverages genetic variants as proxies for the exposure (periodontitis), mimicking the randomization process of RCTs. SNP, single-nucleotide polymorphism.

Mendelian randomization (MR) is a study design that provides an alternative to the hypothetical human RCT requiring the unethical induction of periodontitis in random participants (Fig. 2). Rather than the random allocation of patients to a periodontitis group, this method divides study participants into groups based on the presence or absence of genetic variants strongly associated with periodontitis. This method remains ethical because such genetic variants are assigned randomly when passed from parents to offspring during meiosis according to Mendel’s law of segregation. Studies using MR are becoming more common due to the technique’s ability to overcome the effects of unknown confounders and reverse causation (Davies et al., 2018).

To evaluate the results of studies using MR to identify a causal relationship between periodontitis and ASCVD, we conducted a systematic review following the Preferred Reporting Items for Systematic Reviews and Meta-Analyses guidelines (Page et al., 2021a, Page et al., 2021b). A detailed account of our methods appears in the Supplementary Materials, and a Preferred Reporting Items for Systematic Reviews and Meta-Analyses flow diagram appears in Supplementary Figure 1. Our review identified 22 studies, 4 of which were related to ASCVD (Bell et al., 2020, Li et al., 2024, Ma et al., 2023, Zhou et al., 2021). The study by Zhou et al. (2021) was excluded because it considered periodontitis and dental caries as a single exposure, despite them being genetically distinct diseases. Of the 3 remaining studies, none found evidence of a causal relationship between periodontitis and CAD or stroke (Table 1). The only exception was Ma et al. (2023) who reported a weak causal relationship (odds ratio, 1.052; 95% confidence interval [CI], 1.002-1.104; *P*-value = .042) for the cardioembolic stroke subtype.

**Table 1:** Mendelian randomization

Author, year	Disease/database	SNPs (instrumental variables)	Analysis methods	Results
Bell et al. (2020)	∙ Periodontitis and stroke/MEGASTROKE and UK Biobank (up to 44,221 cases, 424,528 controls)	∙ Periodontitis → stroke, CAD	IVW	There was no robust evidence for a causal relationship between periodontitis and stroke or CAD.
		LOC107984137 (rs729876)	MR-PRESSO	
	∙ Periodontitis and CAD/CARDIoGRAMplusC4D and UK Biobank (122,733 cases, 424,528 controls)	MTND1P5 (rs16870060)	MR-Egger	
		DEFA1A3 (rs2738058)		
		SIGLEC5 (rs4284742)		
		GLT6D1 (rs1537415)		

Ma et al. (2023)	∙ Chronic periodontitis/UK Biobank (950 cases and 455,398 controls)	∙ Chronic periodontitis → ischemic stroke (LAA, SVO, and CE)	IVW	Potential causal effect of chronic periodontitis on cardioembolic stroke.
		15 SNPs	MR-Egger	
	∙ Aggressive Periodontitis/genome-wide association study of European ancestry (851 cases and 6,836 controls)	∙ Aggressive periodontitis → ischemic stroke (LAA, SVO, and CE)	Weighted median	
		9 SNPs		
	∙ Ischemic stroke and its subtypes/MEGASTROKE (34,217 cases and 406,111 controls)			
Li et al. (2024)	∙ Chronic periodontitis/FinnGen (case: 3046, control: 195,395)	∙ CA → periodontitis (chronic and acute)	IVW	No significant causal relationship between CA and periodontitis. Similarly, a bidirectional analysis did not identify any effect from periodontitis on CA.
		29 SNPs	MR-Egger	
	∙ Acute periodontitis/FinnGen (case: 367, control: 195,395)	∙ Periodontitis → CA	Weighted median	
		5 SNPs	Simple mode	
	∙ Coronary atherosclerosis/OpenGWAS (case: 14,334, control: 346,860)		Weighted mode	

ICBP-GWAS, International Consortium of Blood Pressure-Genome-Wide Association Studies; IVW, inverse-variance weighted; SNP, single-nucleotide polymorphism; CAD, coronary artery disease; HF, heart failure; AF, atrial fibrillation; CA, coronary atherosclerosis; LAA, large-artery atherosclerosis; SVO, small-vessel occlusion; CE, cardioembolic.

As outlined in a previous study (van de Luitgaarden et al., 2022), we assessed methodological quality based on criteria representing the key assumptions of MR: whether a full instrumental variable analysis was conducted and whether the 3 MR assumptions were upheld (Supplemental Fig. 2). All the studies we decided to include performed a full instrumental variable analysis using inverse-variance weighting (IVW) as the primary method. Both Bell et al. (2020) and Li et al. (2024) met the first assumption by reporting F-statistics. Ma et al. (2023) did not verify this assumption, however, as they selected genetic instruments with a *P*-value <1 × 10^−5^ without reporting F-statistics. All studies tested the second and third assumptions through various analyses, such as the MR-Egger regression and weighted median methods. None of the studies conducted nonlinear analyses.

Although Ma et al. (2023) found a weak causal relationship between periodontitis and a specific stroke subtype, this must be interpreted with caution because the study violated the first assumption of MR, which requires that the genetic variant be strongly associated with the exposure of interest. Ma et al. (2023) raised the statistical threshold for single-nucleotide polymorphism selection in their study from the conventional genome-wide significance threshold of *P*-value <5 × 10^−8^ to *P*-value <1 × 10^−5^ due to a lack of genetic variants. When the genetic variants used in an MR study are only weakly associated with the exposure of interest, the study is subject to weak instrument bias, increasing the risk of type I errors or false positives (Burgess et al., 2016). Overall, these findings do not support the existence of a causal relationship between periodontitis and ASCVD.

MR studies, while powerful in addressing confounding and reverse causation biases, have several limitations that demand a careful interpretation of their results. One key limitation is that the variance explained by the selected single-nucleotide polymorphisms associated with periodontal disease was relatively low. Although this may reduce the robustness of the findings, weak instrument bias was not a major concern in either of the 2 studies. In addition, because MR relies on the assumption that genetic variants exert a consistent and lifelong influence on exposure, MR studies are more effective for exposures that are stable or chronic over time. MR studies are less appropriate for acute conditions like acute periodontitis because they can fluctuate or resolve quickly. Thus, it is difficult for MR studies to capture their transient nature. Future studies should focus on identifying stronger genetic instruments and consider methods that can also account for shorter exposure lengths, such as those associated with acute periodontitis.

### Common Genetic and Environmental Risk Factors—Confounding Bias

**Fig. 3:**
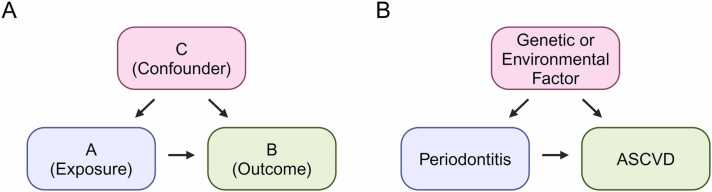
Directed acyclic graphs illustrating confounding bias. (A) A confounder (C) influences both the exposure (A) and the outcome (B). If the confounder is inadequately controlled, it can distort or obscure the association between the exposure and the outcome. (B) Common genetic or environmental factors may act as confounders, distorting the association between periodontitis and ASCVD.

Another plausible explanation for the apparent association between periodontal diseases and ASCVD is the presence of shared genetic or environmental risk factors that act as confounders (Fig. 3). Confounding bias exists when confounder C influences both exposure A and outcome B. This can distort or obscure the association between the exposure and the outcome if the confounding factor is inadequately controlled (Lederer et al., 2019).

*CDKN2B-AS1* (ANRIL) is the best-studied genetic risk factor shared by periodontitis and ASCVD. Located near the *CDKN2A* and *CDKN2B* genes on human chromosome 9p21.3, *CDKN2B-AS1* encodes a long noncoding RNA involved in gene regulation (Hubberten et al., 2019). Several genome-wide association studies (GWAS) identified *CDKN2B-AS1* as being associated with type 2 diabetes, atherosclerosis, and several forms of cancer. Other studies suggested its association with periodontitis (Schaefer et al., 2013, Schaefer et al., 2011, Schaefer et al., 2009). *CAMTA1*/*VAMP3* (Bochenek et al., 2013) and *PLG* (Schaefer et al., 2015) have also been suggested as shared genetic risk factors for both periodontitis and atherosclerosis.

Environmental risk factors such as smoking, oral hygiene, nutrition, and psychological stress can also affect both diseases (Aarabi et al., 2017). Although some of these risk factors are included as covariates when performing association studies, others, such as nutritional status and psychological stress, are often missing from the statistical analyses or are difficult to control (Dietrich et al., 2013). These missing common risk factors may contribute to a false-positive correlation between periodontitis and atherosclerosis.

### Collider Stratification Bias

**Fig. 4:**
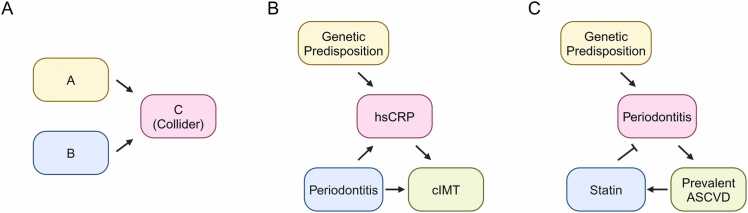
Directed acyclic graphs illustrating collider stratification bias. (A) Collider stratification bias occurs when a collider (C) is influenced by both the exposure (A) and the outcome (B) and is controlled for in the analysis. This can distort the association between A and B, altering the interpretation of the relationship between the exposure and the outcome. (B) When investigating the association between periodontitis (exposure) and carotid intima-media thickness (cIMT) (outcome), high-sensitivity C-reactive protein (hsCRP) acts as a collider because it is influenced by both periodontitis and an unknown genetic predisposition. This introduces bias in identifying an association between periodontitis and cIMT. (C) When investigating the association between periodontitis (exposure) and prevalent ASCVD (outcome), periodontitis may also act as a collider influenced by both statin use and an unknown genetic predisposition. This occurs because severe cases of periodontitis are selected due to the protective effects of statins, creating a spurious relationship between statin use and genetic predisposition. It may falsely appear that statin users with severe periodontitis have a higher genetic predisposition for inflammation, leading to an overestimation of the association between periodontitis and ASCVD.

Collider stratification bias can lead to false-positive associations between periodontitis and ASCVD. This bias occurs when a collider C, influenced by both A and B (which are not necessarily the exposure or outcome), is controlled for in the analysis (Lu et al., 2024) (Fig. 4A). The presence of a collider can suggest an association between A and B where none exists or distorts a true association, leading to misleading conclusions about a causal relationship between exposure and outcome. An excellent example is the analysis conducted by Leite et al. (2020) who looked for collider stratification bias in the association between periodontitis and carotid intima-media thickness, a surrogate marker of ASCVD (Fig. 4B). The authors found a positive association between periodontitis and carotid intima-media thickness (odds ratio: 1.5; 95% CI: 1.1-2.3) using a conventional adjusted logistic regression analysis that included levels of hsCRP as a covariate. The association was lost (odds ratio: 1.3; 95% CI: 0.8–2.0), however, when the method was changed to marginal structural modeling and hsCRP was treated as a constant. In their analysis, hsCRP acted as a collider influenced by both periodontitis and unknown confounders, such as genetic predisposition. This biased the identification of an association between periodontitis and the ASCVD surrogate marker.

Collider stratification bias may also explain the discrepancy in the association between periodontitis and prevalent or incident ASCVD. Retrospective studies investigating the relationship between periodontitis and prevalent ASCVD are particularly susceptible to this form of bias. For example, some medications taken by prevalent ASCVD patients, such as statins, also protect against the effects of periodontitis (Muniz et al., 2018). This would hide the milder cases of periodontitis from detection, supplying only the most severe cases to the study. Consequently, the study then conditions the collider, periodontitis, which is influenced by both statin use and unknown genetic predisposition (Fig. 4C). This creates a spurious relationship between statin treatment and genetic predisposition, making it appear that statin users with severe periodontitis have a higher genetic predisposition for inflammation. In this way, collider stratification bias can lead to an overestimation of the association between periodontitis and ASCVD.

## CONCLUSIONS AND FUTURE DIRECTIONS

Epidemiological research has consistently demonstrated a positive association between periodontitis and ASCVD, but this does not necessarily imply a causal relationship. While periodontal interventions can improve the levels of surrogate markers for ASCVD, the impact of these interventions on actual cardiovascular events remains uncertain. Moreover, MR studies have not confirmed a genetic causality between periodontitis and ASCVD. Positive associations observed in epidemiological studies may also be influenced by biases, such as confounding bias and collider stratification bias. There is also limited support for a causal relationship between periodontitis and ASCVD from animal studies and the biological mechanisms they have so far suggested to underlie such a link.

Future research should focus on long-term RCTs and mechanistic studies to clarify the potential causality. This includes examining molecular pathways where periodontal pathogens like *P gingivalis* and *A actinomycetemcomitans* influence endothelial cells, systemic inflammation, and immune responses through bacterial invasion and molecular mimicry. Animal models that closely mimic chronic human periodontitis, alongside long-term cohort studies in humans, would help determine whether periodontal treatment can prevent cardiovascular events. By integrating findings from molecular, animal, and human studies, the potential causal relationship between periodontitis and ASCVD can be better understood, thereby guiding future therapeutic strategies.

## AUTHOR CONTRIBUTIONS

J.Y. Kim contributed to our literature search, to the generation of figures, and drafted the paper; K. Lee contributed to data interpretation; M.G. Lee reviewed the paper; S.J. Kim contributed to paper conception, data analysis interpretation, and critically revised the paper. All authors gave final approval and agreed to be accountable for all aspects of the work.

## CRediT Authorship Contribution Statement

**June Yeon Kim:** Writing – original draft, data curation. **Kyeongho Lee:** Data curation. **Moon Geon Lee:** Investigation. **Sung-Jin Kim:** Writing – review and editing, validation, supervision, and conceptualization.

## DECLARATION OF COMPETING INTERESTS

The authors declare that they have no known competing financial interests or personal relationships that could have appeared to influence the work reported in this paper.

## Footnotes

Appendix A Supplemental material associated with this article can be found online at doi:10.1016/j.mocell.2024.100146.
