# Supplementary material for: Periodontitis and atherosclerotic cardiovascular disease
Source: Mol Cells. 2024 Nov 6;47(12):100146. doi: 10.1016/j.mocell.2024.100146 (PMC11612374; doi:10.1016/j.mocell.2024.100146)
Supplement: Supplementary file 1 — Supplementary material [file mmc1.docx]

**SUPPLEMENTARY MATERIALS**

**Periodontitis and atherosclerotic cardiovascular disease**

June Yeon Kim^1^, Kyeongho Lee^1^, Moon Geon Lee^1^ and Sung-Jin Kim^1^*

^1^ Department of Oral Histology and Developmental Biology, School of Dentistry and Dental Research Institute, Seoul National University, 101 Daehak-ro, Jongno-gu, Seoul 03080, Korea

^*^Correspondence: e-mail) sjinkim@snu.ac.kr

| **Table of Contents** | **Page** |
| --- | --- |
| Title page | 1 |
| Table of contents | 2 |
| Methods | 3 |
| Supplementary Tables | 6 |
| Supplementary Figures | 7 |
| References | 9 |
|  |  |

##### METHODS

## Protocol

This systematic review was conducted according to the Preferred Reporting Items for Systemic Reviews and Meta-Analyses (PRISMA) guidelines (Page et al., 2021a; Page et al., 2021b).

## Information source and search strategy

To identify relevant published articles, we conducted a search on August 8, 2024, using the Medline, Embase, Web of Science, Scopus, and ScienceDirect databases. The search focused on the relevant MeSH terms 'Periodontitis,' 'Atherosclerosis,' and 'Mendelian randomization.' All authors agreed upon the final search strategies, which are detailed in Supplementary Table 1, along with comprehensive explanations for each database. Additionally, we searched Google and Google Scholar for grey literature to ensure no relevant records were missed.

## Eligibility criteria

We included all Mendelian randomization (MR) studies that provided data on the causal relationship between periodontitis and ASCVD. The eligibility of the identified studies was assessed by thoroughly reviewing the full text. The selected studies performed MR analyses to investigate the causal relationship between periodontitis and ASCVD or coronary atherosclerosis. Additionally, studies that included conditions with distinct etiologies and clinical manifestations, such as dental caries and periodontitis, as exposures or outcomes were excluded.

## Study selection

The corresponding author evaluated the relevance and validity of the initial records retrieved from the search databases. Two authors (J.Y. Kim and K. Lee) independently performed the initial screening and full-text review, as well as a bibliographic review of all included studies. No grey literature was identified from the additional search. Any disagreements or uncertainties regarding study inclusion were resolved through discussions among all authors.

## Data extraction

Two authors (J.Y. Kim and K. Lee) independently extracted information through a full-text review of the included studies. The extracted data included the first author’s name, year of publication, exposure and outcome of interest, data sources, genetic instruments, MR analysis methods, and the main MR analysis results, including odds ratios (ORs), 95% confidence intervals (CIs), and P-values.

## Methodological quality assessment

The methodological quality of the included MR studies was assessed using the approach outlined in a recent systematic review of MR studies (van de Luitgaarden et al., 2022). We first evaluated whether a full instrumental variable (IV) analysis was performed in each study. The assessment was then based on three key assumptions: (1) the genetic variants are associated with periodontitis, (2) the genetic variants are not related to any confounding factors that could influence the periodontitis-outcome relationship, and (3) the genetic variants affect the outcome solely through their association with periodontitis.

## Supplementary Table 1. Search strategies for each database

| **Database** | **Search strategy** |
| --- | --- |
| MEDLINE-Embase* | periodontitis:ab,ti OR periodontitides:ab,ti OR pericementitis:ab,ti OR pericementitides:ab,ti OR 'periodontal disease':ab,ti OR 'periodontal inflammation':ab,ti OR parodontitis:ab,ti OR paradentitis:ab,ti OR 'gum disease':ab,ti OR 'gum inflammation':ab,ti OR gingivitis:ab,ti)  AND  atherosclerosis:ab,ti OR atheroscleroses:ab,ti OR atherogenesis:ab,ti OR atherogeneses:ab,ti OR atherosclerotic:ab,ti OR ascvd:ab,ti  AND  mendelian randomization:ab,ti OR mendelian randomisation:ab,ti  AND  ([article]/lim OR [article in press]/lim) AND [humans]/lim |
| Web of Science | (TI=(periodontitis) OR AB=(periodontitis) OR TI=(periodontitides) OR AB=(periodontitides) OR TI=(pericementitis) OR AB=(pericementitis) OR TI=(Pericementitides) OR AB=(Pericementitides) OR TI=(periodontal disease) OR AB=(periodontal disease) OR TI=(periodontal inflammation) OR AB=(periodontal inflammation) OR TI=(gum disease) OR AB=(gum disease) OR TI=(gum inflammation) OR AB=(gum inflammation) OR TI=(gingivitis) OR AB=(gingivitis) OR TI=(parodontitis) OR AB=(parodontitis) OR TI=(paradentitis) OR AB=(paradentitis))  AND  (TI=(Atherosclerosis) OR AB=(Atherosclerosis) OR TI=(Atheroscleroses) OR AB=(Atheroscleroses) OR TI=(Atherogenesis) OR AB=(Atherogenesis) OR TI=(Atherogeneses) OR AB=(Atherogeneses) OR TI=(atherosclerotic) OR AB=(atherosclerotic) OR TI=(ASCVD) OR AB=(ASCVD))  AND  (TI=(Mendelian Randomization) OR AB=(Mendelian Randomization) OR TI=(Mendelian Randomisation) OR AB=(Mendelian Randomisation)) |
| Scopus | TITLE-ABS-KEY(periodontitis) OR TITLE-ABS-KEY(periodontitides) OR TITLE-ABS-KEY(pericementitis) OR TITLE-ABS-KEY(Pericementitides) OR TITLE-ABS-KEY(periodontal disease) OR TITLE-ABS-KEY(periodontal inflammation) OR TITLE-ABS-KEY(gum disease) OR TITLE-ABS-KEY(gum inflammation) OR TITLE-ABS-KEY(gingivitis) OR TITLE-ABS-KEY(parodontitis) OR TITLE-ABS-KEY(paradentitis)  AND  TITLE-ABS-KEY(Atherosclerosis) OR TITLE-ABS-KEY(Atheroscleroses) OR TITLE-ABS-KEY(Atherogenesis) OR TITLE-ABS-KEY(Atherogeneses) OR TITLE-ABS-KEY(atherosclerotic) OR TITLE-ABS-KEY(ASCVD)  AND  TITLE-ABS-KEY(Mendelian Randomization) OR TITLE-ABS-KEY(Mendelian Randomisation) |
| ScienceDirect | (periodontitis OR pericementitis OR gum OR periodontal)  AND  (atherosclerosis OR atherosclerotic)  AND  (Mendelian randomization OR Mendelian randomisation) |

* The Embase database was utilized for the search as it encompasses the search conducted in MEDLINE (Lam et al., 2018).

## Supplementary Figure 1. PRISMA 2020 flow diagram for new systematic reviews that include searches of databases, registers, and other sources.


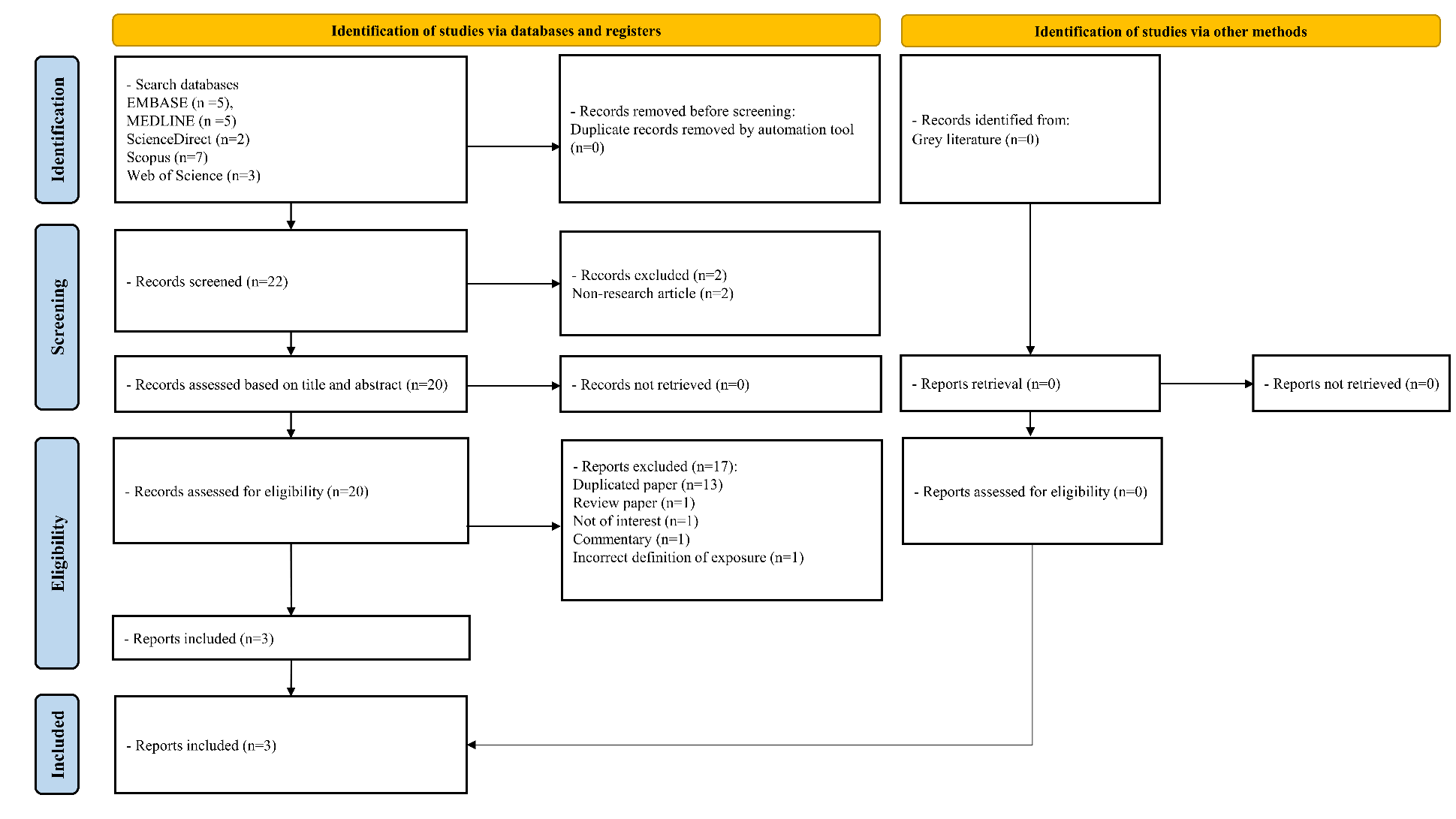


## Supplementary Figure 2. Methodological quality assessment of included Mendelian randomization studies.


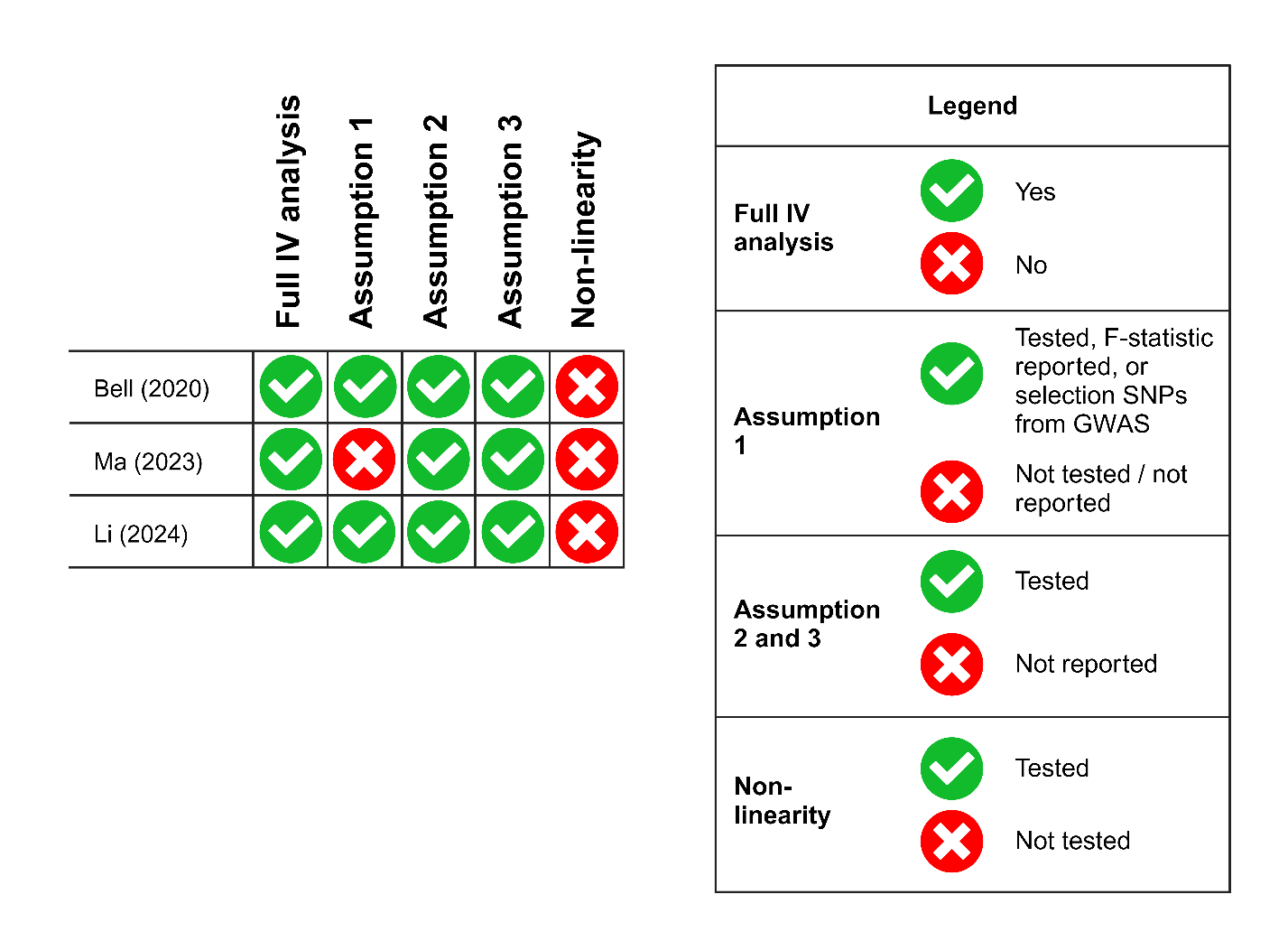


The methodological quality of Mendelian randomization studies was assessed and sorted by first author name and publication year. The evaluation considered whether a full IV analysis was performed and was based on three key assumptions: (1) the genetic variants are associated with periodontitis, (2) the genetic variants are not associated with any confounders that could influence the periodontitis-outcome relationship, and (3) the genetic variants affect the outcome only through their association with periodontitis.

**References**

Lam, M.T., De Longhi, C., Turnbull, J., Lam, H.R., and Besa, R. (2018). Has Embase replaced MEDLINE since coverage expansion? Journal of the Medical Library Association: JMLA 106, 227.

Page, M.J., McKenzie, J.E., Bossuyt, P.M., Boutron, I., Hoffmann, T.C., Mulrow, C.D., Shamseer, L., Tetzlaff, J.M., Akl, E.A., and Brennan, S.E. (2021a). The PRISMA 2020 statement: an updated guideline for reporting systematic reviews. Systematic reviews 10, 1-11.

Page, M.J., Moher, D., Bossuyt, P.M., Boutron, I., Hoffmann, T.C., Mulrow, C.D., Shamseer, L., Tetzlaff, J.M., Akl, E.A., and Brennan, S.E. (2021b). PRISMA 2020 explanation and elaboration: updated guidance and exemplars for reporting systematic reviews. bmj 372.

van de Luitgaarden, I.A.T., van Oort, S., Bouman, E.J., Schoonmade, L.J., Schrieks, I.C., Grobbee, D.E., van der Schouw, Y.T., Larsson, S.C., Burgess, S., van Ballegooijen, A.J., et al. (2022). Alcohol consumption in relation to cardiovascular diseases and mortality: a systematic review of Mendelian randomization studies. Eur J Epidemiol 37, 655-669.
